# Cambrian suspension-feeding tubicolous hemichordates

**DOI:** 10.1186/s12915-016-0271-4

**Published:** 2016-07-07

**Authors:** Karma Nanglu, Jean-Bernard Caron, Simon Conway Morris, Christopher B. Cameron

**Affiliations:** Department of Ecology and Evolutionary Biology, University of Toronto, Toronto, Ontario M5S 2J7 Canada; Department of Natural History Palaeobiology, Royal Ontario Museum, Toronto, Ontario M5S 2C6 Canada; Department of Earth Sciences, University of Cambridge, Cambridge, CB2 3EQ UK; Département de sciences biologiques, Université de Montréal, Montréal, PQ H2V 2S9 Québec Canada

**Keywords:** Enteropneusta, Hemichordata, Cambrian, Burgess Shale

## Abstract

**Background:**

The combination of a meager fossil record of vermiform enteropneusts and their disparity with the tubicolous pterobranchs renders early hemichordate evolution conjectural. The middle Cambrian *Oesia disjuncta* from the Burgess Shale has been compared to annelids, tunicates and chaetognaths, but on the basis of abundant new material is now identified as a primitive hemichordate.

**Results:**

Notable features include a facultative tubicolous habit, a posterior grasping structure and an extensive pharynx. These characters, along with the spirally arranged openings in the associated organic tube (previously assigned to the green alga *Margaretia*), confirm *Oesia* as a tiered suspension feeder.

**Conclusions:**

Increasing predation pressure was probably one of the main causes of a transition to the infauna. In crown group enteropneusts this was accompanied by a loss of the tube and reduction in gill bars, with a corresponding shift to deposit feeding. The posterior grasping structure may represent an ancestral precursor to the pterobranch stolon, so facilitating their colonial lifestyle. The focus on suspension feeding as a primary mode of life amongst the basal hemichordates adds further evidence to the hypothesis that suspension feeding is the ancestral state for the major clade Deuterostomia.

**Electronic supplementary material:**

The online version of this article (doi:10.1186/s12915-016-0271-4) contains supplementary material, which is available to authorized users.

## Background

Hemichordates are central to our understanding of deuterostome evolution. The two classes (tubicolous Pterobranchia and vermiform Enteropneusta) are monophyletic [1–3], but are morphologically disparate (however, see [4, 5] for an alternate viewpoint of Pterobranchia as sister to the family Harrimaniidae within a paraphyletic Enteropneusta). Accordingly they give only generalized clues as to both the anatomy and mode of life of the last common ancestor as well as its connections to the sister phylum Echinodermata (collectively Ambulacraria). The resistant tubaria of pterobranchs (notably the Paleozoic graptolites [6]) provide an adequate fossil record, but in contrast that of the enteropneusts is almost non-existent [7–9]. One exception is a tubicolous taxon (*Spartobranchus tenuis*) from the middle Cambrian Burgess Shale [10]. This enteropneust is closely comparable to extant harrimaniids, although its organic tube finds no modern counterpart [11]. The coeval *Oesia disjuncta* Walcott [12] has been compared to groups as diverse as annelids [12], appendicularian tunicates [13] and chaetognaths [14, 15], thus remaining in phylogenetic limbo. The proposed chaetognath affinity was refuted by Conway Morris [16] and a hemichordate affinity briefly suggested instead, albeit without detailed re-observation of original specimens or consideration of new material. On the basis of hundreds of specimens from the newly discovered Marble Canyon fossil locality (Kootenay National Park, British Columbia) [17], we not only identify *Oesia* as a primitive enteropneust but also demonstrate that it constructed the perforated tube-like fossils previously assigned to *Margaretia dorus* and interpreted as thalli of a green alga similar to *Caulerpa* [18].

## Results

**Fig. 1:**
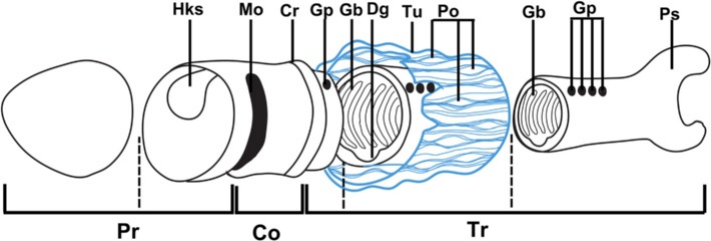
Schematic anatomy of *Oesia disjuncta*. Co: collar, Cr: circum-collar ridge, Dg: digestive groove, Pr: proboscis, Hks: heart-kidney-stomochord complex, Gb: gill bars, Gp: gill pores, Mo: mouth, Po: pores, Ps: posterior structure, Tr: trunk, Tu: tube. *Dashed lines* indicate transverse cross sections

**Fig. 2:**
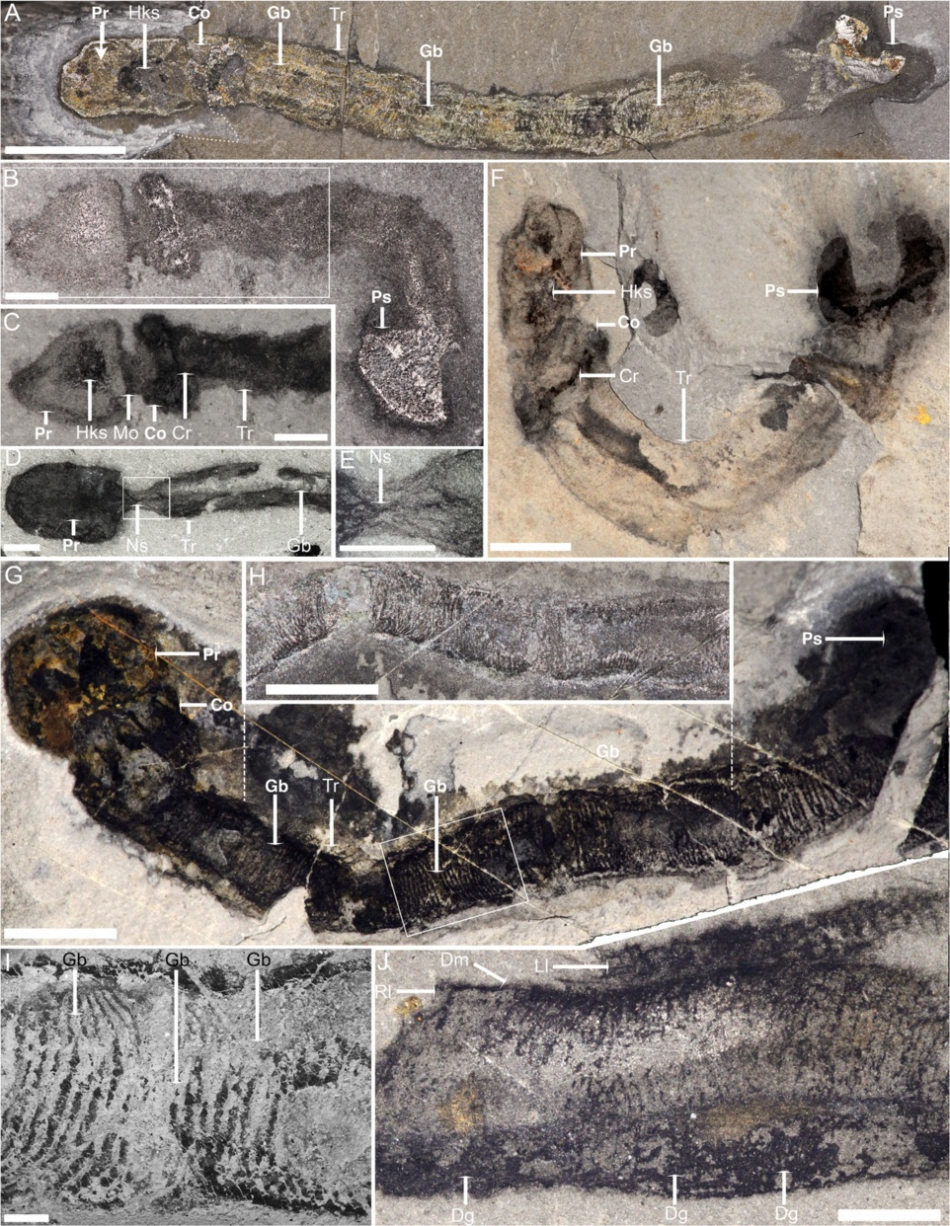
General morphology of *Oesia disjuncta* from the Burgess Shale. (Specimens in **d**, **e** and **j** come from the Walcott Quarry; all other specimens come from Marble Canyon)*.* **a** Note bilobed posterior structure and extended pharyngeal area (ROM 63737, part and counterpart are superimposed at the *dashed line*). **b**, **c** Tripartite body plan and internal organs in the proboscis (ROM 63711). **d**, **e** Large proboscis and possible nuchal skeleton (USNM 509815), see also Additional file 5A–C. **f** Well-developed bilobed posterior structure (ROM 63713). **g**–**i** Details of the pharyngeal area (**h**, partial counterpart of **g**, highlighted by *vertical dashed line*; **i** is close-up of framed area in **g**, ROM 63710). **j** Left and right pairs of gill bars preserved in lateral view (USNM 277844). Direct light images: **a, b**, **h**; polarized light images: **c**–**g**, **j**; SEM image: **i**. Co: collar, Cr: circum-collar ridge, Dg: digestive groove, Dm: dorsal midline, Gb: gill bars, Hks: heart-kidney-stomochord complex, Ll: lateral side left, Lr: lateral side right, Ns: nuchal skeleton, Pr: proboscis, Ps: posterior structure, Tr: trunk. Scale bars: **a** = 10 mm, **b**–**e** = 1 mm, **f**–**h** = 5 mm, **i** = 500 μm, **j** = 2 mm

*Oesia* possesses the canonical enteropneust body plan of proboscis, collar and elongate trunk (Figs. 1, 2a, b) but is unusual in that posterior to the pharynx there is a bilobed structure, rather than a vermiform intestine. Body length averages 53 mm (*n* = 187, size range 2.4–120 mm), but the width seldom exceeds 10 mm. The proboscis is relatively elongate (ratio of length to width is 1.35 ± 0.58) and variable in shape (Figs. 2a, d, e, g, h; Additional files 1A, D–F, 2A, F–I, 3C, 4). A conspicuous ovoid area at the medial base of the proboscis appears darker or more reflective than the surrounding area (Fig. 2a–c, f; Additional files 2F–I, 3C). This is interpreted as the heart-kidney-stomochord complex [11]. More irregular structures across the proboscis probably represent decayed musculature (Fig. 2c; Additional file 2F–I). The collar is rectangular, but with rounded edges (Fig. 2a–c, f, g; Additional files 1A, D–F, 2F–I, 3C, 5D, E, G). In proportion it is shorter than the proboscis (average ratio is 0.39 ± 1.12) but has an equivalent width (average proboscis to collar width is 1.08 ± 0.23 mm). At the posterior margin of the collar (Fig. 2b, c, f; Additional file 2F–I), a dark or reflective band probably represents the circum-collar ridge, while a thin, longitudinal structure between the proboscis base and collar (Fig. 2d, e; Additional file 5A, B, D–I) is interpreted as the nuchal skeleton. The pharyngeal region houses a series (about 3 bars/mm) of approximately U-shaped gill bars (Fig. 2g–j; Additional file 5A, C) but is remarkable in that it occupies approximately 80 % of the trunk length (Fig. 2a, g; Additional files 2A, C, 5D, E, G). The posterior end of the trunk is bulbous (Fig. 2b, g; Additional files 1D–E, 2H, 4, 5D, E, G) and sometimes terminates in a bilobed structure (Fig. 2a, f; Additional files 1B, C, 2A–D, F, H) that is usually wider than long (average width-to-length ratio is 1.48 ± 0.63).

**Fig. 3:**
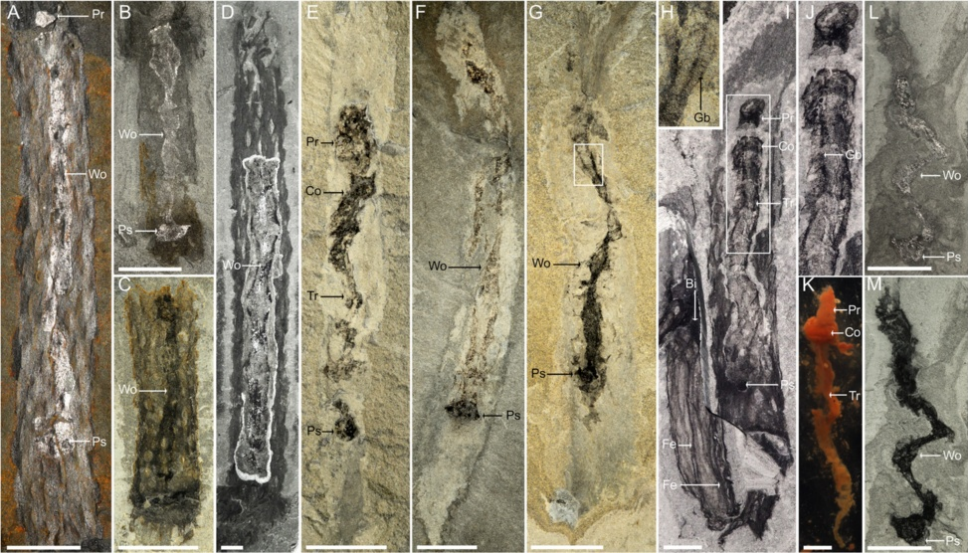
*Margaretia dorus* tubes and associations with *Oesia disjuncta* from the Burgess Shale. Specimens in **a** and **d** come from the Raymond Quarry; all other specimens come from Marble Canyon*.* (**a**–**h**) Taphonomic gradient of the worm inside its tube from generally poorly preserved (**a**) to better preserved (**h**); the tubes tend to preserve more poorly at Marble Canyon relative to tubes from the Raymond Quarry showing similar amounts of decay of the worm. **a** Holotype of *M. dorus* with worm preserved as a dark/reflective band along the central axis of the tube (USNM 83922). **b**, **c** Small fragments of tubes containing worms showing only few recognizable features (**b**: ROM 63955, **c**: ROM 63956). **d** Part of a tube excavated to reveal a poorly preserved worm inside (ROM 63715). **e** Tripartite body plan recognizable but worm heavily decayed (ROM 63953). **f** Clear posterior structure but indistinct proboscis and trunk (ROM 63957). **g** Poorly preserved trunk and faded tube (ROM 63952). **h** Close-up of framed area in **g** on counterpart, showing gill bars readily visible. **i**, **j** Specimen showing clear tripartite body plan and evidence of gill bars (ROM 63715). **k** The extant acorn worm *Saccoglossus pusillus* after 48 hours of decay at 25 °C showing dissociated parts, although the tripartite body plan is still recognizable. **l**, **m** *O. disjuncta* outside of its tube, showing extreme signs of decay comparable with **k**. Direct light (**l**) is contrasted with polarized light (**m**) to reveal different aspects of fossil morphology (ROM 63954). The ectoderm is fraying off, the proboscis is indistinct and the trunk has lost turgidity. Most worms preserved inside their tubes show a similar level of preservation. Direct light images: **a**, **b**, **d**, **l**; polarized light images: **c**, **e**–**i**, **m**. Bi: node of bifurcation, Fe: fibrous elements, Wo: worm, other acronyms see Figs. 1 and 2. Scale bars: **a**–**c**, **e**–**g**, **k**–**m** = 10 mm, **d**, **i** = 5 mm

**Fig. 4:**
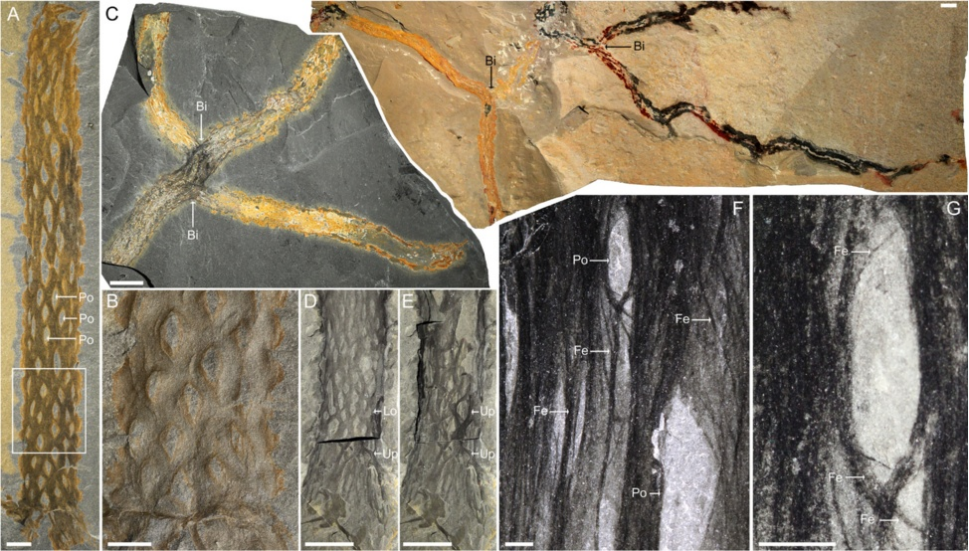
**a**, **b** Spirally arranged pores perforate the tube (ROM 63716; see also Additional file 9A). **c** Two examples of multiple bifurcation points in a single specimen. Extreme size variation underscores the fragmentary nature of most tubes (*left*: KUMIP 204373, *right*: KUMIP 241392). **d**, **e** Tube showing three-dimensional preservation. **d** Large section of the tube has been broken off revealing the other side of the tube. **e** The broken segment has been placed back in its original configuration to illustrate the three-dimensionality of the tube (KUMIP 147911). **f**, **g** Close-up of the pores and fibrous texture of the tube. Individual fibres are micrometre small (ROM 63705). Bi: node of bifurcation, Fe: fibrous elements, Lo: lower surface, Po: pores, Up: upper surface, Wo: worm, other acronyms see Fig. 2. Scale bars: **a**, **b**, **f**, **g** = 5 mm, **c**–**e** = 10 mm

The preservation of *Oesia* (*n* = 45 in Marble Canyon, *n* = 6 in Raymond Quarry) inside *Margaretia* (now a junior synonym of *Oesia disjuncta*) suggests an original association (Fig. 3a; Additional file 6). Only single worms are found within tubes, suggesting a solitary mode of life, although due to breakage during transport, it is conceivable that tubes may have been inhabited by more than one worm (Fig. 3b–d). Typically the tube is at least twice the width of the worm, suggesting the worm could move freely within its dwelling (Fig. 3e–j; Additional files 3B, 7, 8). Three-dimensional preservation of both sides of the tube (Fig. 4d, e) shows that the internal cavity of the tube was spacious, and that the tube was at least semi-rigid. The total length and extremities of the tubes are poorly known. This is because of either prior breakage or concealment (Fig. 4a), but at least one end (presumably the top of the tube) appears rounded and closed (Additional file 9B, C).

Tubes with irregular undulations and lacking the spiral pattern were previously interpreted as prostrate subterranean rhizomes (Fig. 4.2-3 [18]). While the reassignment from alga to organically produced tube invalidates this identification, it remains plausible that subterranean, lateral extensions of the tube could serve as an anchor. In any individual the width of the tube is usually consistent along the length, but otherwise it varies considerably (4–20 mm). Occasionally a tube shows one (Fig. 3i; Additional files 7A, C, 8A–C, 9D–F) or, more rarely, two bifurcations (Fig. 4c). Each bifurcates at approximately the same angle and has the same width as the primary tube. The tube wall is perforated by spirally arranged pores (about 10 openings per revolution; Fig. 4a, b). In a single tube pore size varies. Some may be almost closed, but others have diameters equivalent to about a third of the tube width (Fig. 4a, b, d, e; Additional file 9A). Pore shape varies from circular to oblong ellipse and rhombic. That these might simply be taphonomic variations is less likely given that the specimens are preserved parallel to the bedding plane (Fig. 4a, b, g–e; Additional file 9A). The margins of the pores tend to be raised, imparting a semi-corrugated texture to the external surface of the tube (Fig. 4a, b; Additional file 9A). The tube is composed of narrow fibres (about 7 μm) that are braided and/or overlain in bundles (Fig. 4f, g).

*Margaretia dorus* is unlike any known species of Paleozoic algae. In particular, the combination of a fibrous composition and elaborate pore architecture are inconsistent with an algal grade of organization, as are its biotic associations and size in relation to well-established Cambrian macroalgae [19]. This in turn argues against *Oesia* being an example of inquilinism. While the dozens of co-occurrences of *O. disjuncta* and its tube strongly suggest an original association, the preservation of large numbers of isolated *Oesia* specimens on single bedding surfaces (Additional files 3, 4) at Marble Canyon also needs an explanation. One possibility is that the association was facultative and *Oesia* could alternate between a tubicolous and non-tubicolous existence. Alternatively the worm may have been forced to vacate the tube as an en masse evacuation prior to final burial. This may be related to both the high-energy burial events [17] and the resultant dysoxic conditions that such events create [20], although this hypothesis is weakened by the lack of obvious exit structures (i.e. there is no evidence the worms could enter or leave the tubes at either end).

In this context, fragmentation of the tubes and dispersal during transport is perhaps a more plausible explanation as to how the worms became isolated. This appears to be reasonable given the observation that although tubes with a length of up to 544 mm are known (Fig. 4c), tubes of comparable width can be not only significantly shorter (e.g. Figs. 3b, 4c), but sometimes are even smaller than the worms themselves. A related observation is that along the tube margins showing evidence for breakage, the bundles of fibres may exhibit a pattern of ’unbraiding.’ This suggests that originally the tubes were vulnerable to damage (Fig. 3b).

The second factor is that in at least some cases the tube evidently serves to conceal the worm. For a worm to be readily visible, the tube either needs to be prepared mechanically, split more or less along the axis or be sufficiently degraded so as to allow a view of the interior. Accordingly, tubes showing such evidence of degradation also contain worms in an evident state of decay (Fig. 3b–h). In such cases worms are poorly preserved and are effectively reduced to a narrow band of reflective carbon (Fig. 3k–m). Worms in such late stages of decay also show a tendency to bend at sharp angles into semi-discrete sections (Figs. 2g, 3e, f, l, m). This appearance may represent adjacent sets of gill bars maintaining their articulation through attachment to the collagenous basal lamina, but at points where this basal lamina has degraded, the more acutely angled bending occurs [11].

## Discussion

Establishing *Oesia* as an enteropneust that inhabited the tube previously identified as the alga *Margaretia* has significant implications for the Cambrian paleogeography and paleoecology of this group. Until now, *Oesia* was one of the rarest of Burgess Shale taxa and was restricted to the Walcott Quarry [21]. At the coeval Marble Canyon locality, however, it is amongst the five most abundant taxa [17] and occupied a key trophic position. In marked contrast, *Margaretia* is recorded from various Burgess Shale sites in Laurentia (including the Stephen Formation of British Columbia and the Spence and Wheeler Shales of Utah [18] — Additional file 11: Table S1), eastern Yunnan, China [22] and further afield in Siberia (originally referred to as *Aldanophyton* [18]) [23]. This expanded distribution suggests that enteropneusts were a significant component of many Cambrian communities (Additional file 6, Additional file 11: Table S1).

**Fig. 5:**
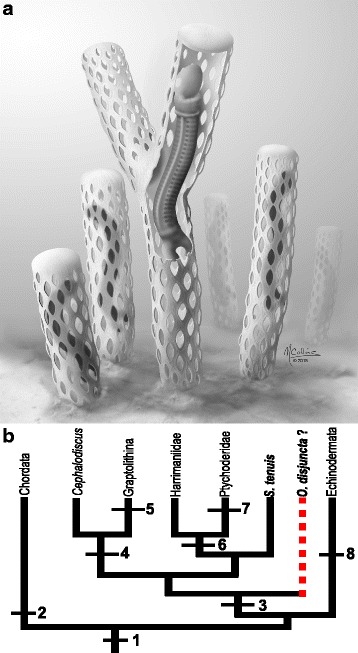
**a** Life reconstruction with hypothetical closed terminal ends of the tubes — part of one tube partially removed to show a worm (drawing by Marianne Collins). **b** Phylogenetic relationship of Deuterostomia derived from [2]. Mapping of characters based on [1, 2] with our proposed hypothetical position for *Oesia disjuncta* as a basal hemichordate (*dashed line with question mark*). The position of *Spartobranchus tenuis* is based on a taphonomic study of extant and fossil enteropneusts [11]. Character states: 1) pharyngeal gill bars, suspension feeding; 2) notochord; 3) tubicolous; 4) miniaturization, coloniality; 5) fuselli; 6) loss of tubicolous lifestyle, deposit feeding; 7) indirect development via tornaria larva; 8) stereom, water vascular system

*Oesia* also throws important new light on the early evolution of the hemichordates. Construction of a large, complex and presumably metabolically costly tube is consistent with a sessile lifestyle. Given that pores appear to be present on all sides, we suggest that the tube (and branches) stood vertically, with the basal region embedded in the substrate and the top presumably closed (Fig. 5a). The porosity of the tube would have prevented dysoxia and also allowed access to the water column for filter feeding. Given that the tubes could exceed 50 cm (Fig. 4c), this suggests a tiering level at least equivalent to (if not above) the tallest sponges known from the Burgess Shale.

The strikingly extended pharynx and numerous gill bars that were employed in suspension feeding are functionally convergent with the hyperpharyngotremy seen in the tunicates [24], cephalochordates [25] and some Paleozoic jawless fish [26] (Fig. 5a). More generally, however, the pharyngeal arrangement seen in *Oesia* suggests that within the hemichordates as a whole suspension feeding was primitive. Whilst a few members of the basal harrimaniids facultatively filter interstitial pore water [27, 28], in extant enteropneusts the primary mode is deposit feeding, consistent with their mostly infaunal existence. Such a migration from an epifaunal existence may have been in response to increased predation pressure and as a consequence entailed significant anatomical changes. Notably in *Oesia* the post-pharyngeal trunk appears to lack the esophageal organ, which in extant forms serves to remove excess water from the food cord (and presumably performed the same function in *Spartobranchus tenuis* where it is also present; a summary of the main differences between *S. tenuis* and *O. disjuncta* can be found in Additional file 10). So too in the more derived taxa the hepatic caeca increase the absorptive area, presumably reflecting the increased demands of deposit feeding.

*Oesia* shares with the co-eval tubicolous *S. tenuis* [10] a bulbous posterior structure which may have acted as an anchor. In *Oesia*, however, the claw-like arrangement points to a more active role in attachment and release, perhaps as a consequence of its inhabiting a commodious tube. This interpretation draws potential comparisons to the juvenile post-anal tail of harrimaniid enteropneusts. This tail serves in ciliary locomotion and as an attachment device and may also be the homologue of the pterobranch stalk [27]. In this context, the specialized posterior structures seen in *S. tenuis* and *O. disjuncta* may actually be ancestral features. If so, these were ultimately lost in the crown group Enteropneusta, but in the Pterobranchia they helped to pave the way towards coloniality.

## Conclusions

While too few morphological characters are available to permit a meaningful cladistic analysis, the unique combination of characters found in *O. disjuncta* encourages us to present a preliminary re-interpretation of early hemichordate evolution. First, a tubicolous, epifaunal and solitary habit are evidently primitive. The fibrous filaments of the *Oesia* tube have some resemblance to the fusellar fibres seen in graptolites such as the Cambrian *Mastigograptus* [29], as well as the comparable periderm of rhabdopleurid [30] and cephalodiscid pterobranchs [31]. An important inference is that *Oesia* (and *Spartobranchus*) possessed secretory glandular cells, presumably homologous with those located on the cephalic shield of the pterobranchs. The apparent absence of fibres in the tubes of *Spartobranchus* suggests that their loss may have preceded the loss of the tube itself. Concurrent with a shift to a burrowing and deposit feeding existence, the crown group enteropneusts abandoned the construction of such tubes. In contrast, the tubes of pterobranchs (and correspondingly the posterior stalk) were elaborated in parallel with their miniaturization and sessile coloniality (Fig. 5b). Crucially, the unique mix of pterobranch and acorn worm characteristics seen in *Oesia* suggests that an extensive pharynx and undifferentiated trunk are basal to the hemichordates, whereas *Spartobranchus* is more derived and is basal to the acorn worms [11]. Future discoveries of new Cambrian hemichordates will help elucidate the hypothesized transformation of the posterior structures into the pterobranch stolon and critically unveil the order of both trait acquisition and loss during the early diversification of this phylum.

Finally, the evidence that primitive enteropneusts were suspension feeders is congruent with the hypothesis that suspension feeding represents the primitive mode of life in deuterostomes [32] as a whole. In particular, it is notable that this lifestyle is seen in early stem-group echinoderms [33] and stem-group ambulacrarians [34], and is inferred in the ur-ambulacrarians [35] as well as the more problematic vetulicolians [36] and yunnanozoans [37].

## Methods

Sediment overlaying sections of some specimens was removed using a micro-engraving tool with a carbide bit. Specimens were observed using a stereomicroscope and photographed using different illuminations, using direct or cross-polarized light on dry or wet specimens. Backscatter scanning electron images were obtained to visualize fine anatomical features. Measurements of morphology were made using the program ImageJ. A list of specimens used in the analysis can be found in Additional file 11: Table S1 [38–47].

## Additional files

Additional file 1: High-resolution imagery of the original three *Oesia disjuncta* specimens figured by C.D. Walcott, 1911, from the Burgess Shale (Walcott Quarry). (A–C) Lectotype (A, part; B, C, counterpart). The serial striations throughout the trunk initially lead Walcott to place this animal amongst the Annelida. They are re-interpreted as gill bars throughout an extended pharynx. This specimen also shows the posterior bilobed structure (USNM 57630). (D, E) The proboscis, collar, trunk and gill bars are all apparent (USNM 57631). (F) The proboscis, circum-collar ridge and gill bars are extremely pronounced (USNM 57632). Direct light images: A, C, D, F; polarized light images: B, E. For acronyms, see Fig. 1. Scale bars: 10 mm. (JPG 8141 kb) Additional file 2:
*Oesia disjuncta* from the Burgess Shale (all specimens from Marble Canyon except USNM 203001, 203033 (C–E – Walcott Quarry). (A–B) Specimen showing the proboscis, gill bars, and exceptional preservation of the posterior bi-lobed structure (B = close-up of framed area in A) (ROM63714). (C–E) Nearly complete specimen — anterior region (to the left) missing; pharyngeal gill bars extending almost completely to the terminal end of the trunk and bilobed posterior structure (D, E = close-ups of framed areas in C) (USNM 203001, 203033). (F–G) Complete specimen showing rounded proboscis, collar, gill bars and bi-lobed posterior structure (ROM 63709). (H, I) Complete specimen showing rounded proboscis, kidney-heart-stomochord complex, collar, circum-collar ridge, gill bars and posterior structure. A patterning texture in the proboscis suggests possible proboscis muscles (ROM63707). Direct light images: A, F, H; Polarized light images: C, D, E, G, I. For acronyms see Fig. 1. Scale bars: A, C, F, G, =5 mm; B, D, E=1mm; H=10 mm; I=2 mm. (PNG 60109 kb) Additional file 3: Cluster of *Oesia disjuncta* from the Burgess Shale (Marble Canyon) preserved on the surface of one large slab (ROM 63735). *O. disjuncta* is highly gregarious at the Marble Canyon, occurring in high abundance across all stratigraphic levels (see also Additional file 7). (A) Overall slab. (B–C) Close-ups of framed areas in A. The specimen on the left in B is preserved in a decayed tube. The specimen in C shows tripartite body plan, gill bars and kidney-heart-stomochord complex. Polarized light images: A–C. Acronyms see Fig. 1. Scale bars: A = 10 cm; B, C = 1 cm. (PNG 59904 kb) Additional file 4: Close up of a cluster of *Oesia disjuncta* from the Burgess Shale (Marble Canyon) preserved on the surface of one large slab (ROM 63736). Specimens show clear tripartite body plan as well as variation in proboscis size and shape. Direct light image: A; Polarized light image: B. For acronyms, see Fig. 1. Scale bars = 1 cm. (PNG 61235 kb) Additional file 5: Previously un-figured *Oesia disjuncta* specimens from the Burgess Shale Walcott's Quarry – Smithsonian Institution collection. (A–C): Partially dissociated specimen showing nuchal skeleton and gill bars (B and C = close ups of framed areas in A) (USNM 509815) – see also Fig. 1d–e. (D–F) Complete specimen with proboscis slightly dissociated from the rest of the body. The thin element at the posterior of the proboscis is likely the nuchal skeleton. Gill bars are also visible, and the posterior structure is preserved laterally (F = close up of framed area in E) (USNM 202440). (G–I) Complete specimen possibly showing the nuchal skeleton (USNM 202145, 203031). Direct light images: D, G; Polarized light images: A–C, E, F, H, I. For acronyms, see Fig. 1. Scale bars: A, D, E: 5 mm; B, C: 1 mm; F, I: 2.5 mm; G, H: 10 mm. (PNG 69939 kb) Additional file 6: Stratigraphic variation in abundance of *Oesia disjuncta* and *Margaretia dorus* in the Marble Canyon paleocommunity. Bars and diamonds indicate numerical abundances of each taxon across 10 cm stratigraphic bins. Coloured bars indicate the percentage of the total number of specimens found within that bin from *O. disjuncta* and *M. dorus* (total community size estimates from 2012 and 2014 field collections). Numbers next to the bars indicate the number of occurrences of *Oesia* preserved inside of *Margaretia* observed within that bin. Stratigraphic levels on the vertical axis represent negative meters from the boundary between the Eldon and Stephen Formations as a reference point. (PNG 62 kb) Additional file 7: Detailed imagery of ROM 63738 (see also Fig. 2d, e). Reflective areas in direct light tend to appear black using polarized light, emphasizing for example the kidney-heart-stomochord complex and the posterior structure (B, D are close-ups of framed areas in A and C). Direct light images: A, B; polarized light images: C, D. For acronyms see Fig. 1 and Fig. 2. Scale bars: A, C: 10 mm; B, D: 5 mm. (PNG 54067 kb) Additional file 8: Additional specimens of *Oesia disjuncta* preserved inside branching tubes from the Burgess Shale (Marble Canyon). The worms show a typical high degree of reflectivity in contrast with the surrounding tubes. (A, B) ROM 63712. (C–E) ROM 63708. Direct light images: A, D; polarized light images: B, C, E. Br1: branch 1, Br2: branch 2, other acronyms see Fig. 1 and Fig. 2. Scale bars: A, B, E: 5 mm, C, D = 10 mm. (PNG 64717 kb) Additional file 9: Morphology of *Margaretia dorus* from the Burgess Shale (Raymond Quarry: A, C, D; Marble Canyon: E; Utah: B; Monarch Cirque: F). (A) Close-up of terminal end showing evidence of limited breakage and variability in pore sizes and shapes. The margins of the pores are upraised, giving the tube a semi-corrugated appearance when viewed laterally (ROM 911390). (See also Fig. 3a.) (B) Two specimens from the Spence Shale with possible rounded terminal ends (see *arrows*) (ROM 59635). (C, D) Specimen showing a central hole at one end, presumably representing the insertion of a more or less perpendicular branching tube (ROM 63739). (E) Specimen showing a circular structure corresponding to a node of bifurcation similar to ROM 63739 (ROM 63706), with a possible rounded terminal end (see *arrow*). (F) Complete specimen also illustrated in Fig. 3g–h (framed area see Fig. 3h). This branching tube shows that pore density decreases near the node of bifurcation, that pore shape varies from rhomboid to more ellipse shaped, and that individual fibres are long and continuous through large sections of the tube (ROM 63716). Direct light images: A, B, E; polarized light images: C, D, F. Te: terminal end; for other acronyms, see Fig. 3. Scale bars: A, E: 5 mm, B, C, D, F: 10 mm. (PNG 68915 kb) Additional file 10: Major morphological differences between the two Cambrian, tubicolous enteropneusts *Spartobranchus tenuis* (*top box*) and *Oesia disjuncta* (*middle box*): (1) *S. tenuis* is thin and elongate and the trunk much more variable in width compared to *O. disjuncta*, which is stout and does not vary in width across the length of the trunk; (2) the pharyngeal gill bars are restricted to approximately 10–20 % of the total trunk length [10] in *S. tenuis*, but extend approximately 80 % of the total trunk length in *O. disjuncta*; (3) *S. tenuis* possesses an esophageal organ while *O. disjuncta* does not; (4) *S. tenuis* has a bulbous terminal structure while *O. disjuncta* has a claw-shaped terminal apparatus; also the tube of *S. tenuis* has an externally corrugated but smooth texture with no evidence of pores or openings (*bottom box*; ROM 94189, while the tube of *O. disjuncta* is fibrous, much larger and has helicoidally arranged openings of variable sizes (not illustrated in this figure). Scale bars for the line drawings: 1 cm, scale bars for the tube: 1 mm. (PDF 7926 kb) Additional file 11: Table S1. Biogeographical distribution of the fossil *Margaretia* and index of specimens used in this study. Reference numbers can be found in the main text. (XLSX 13 kb)
