# Supplementary figures and images for: Cambrian suspension-feeding tubicolous hemichordates
Source: BMC Biol. 2016 Jul 7;14:56. doi: 10.1186/s12915-016-0271-4 (PMC4936055; doi:10.1186/s12915-016-0271-4)

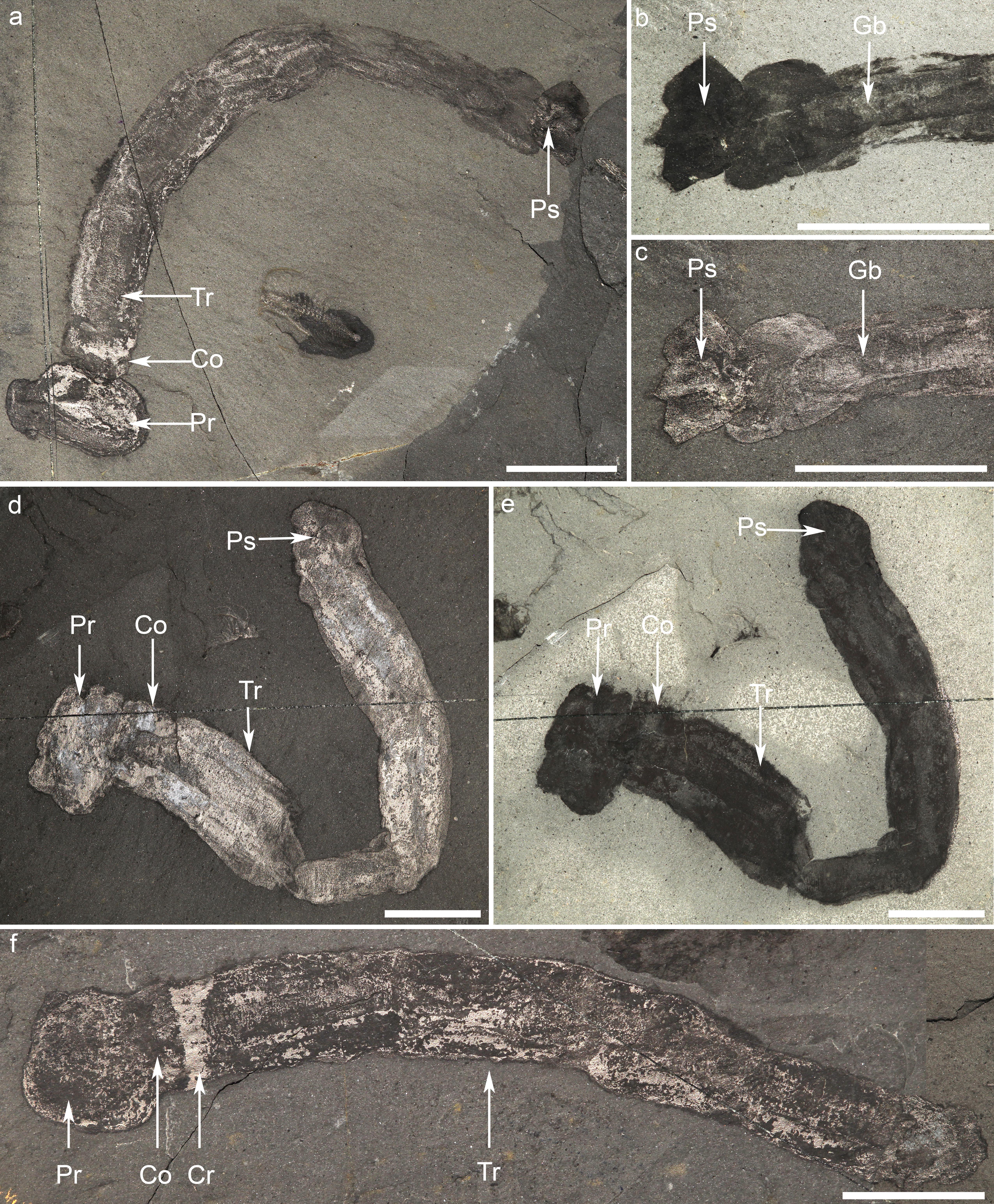

Supplement: Additional file 1: — High-resolution imagery of the original three Oesia disjuncta specimens figured by C.D. Walcott, 1911, from the Burgess Shale (Walcott Quarry). (A–C) Lectotype (A, part; B, C, counterpart). The serial striations throughout the trunk initially lead Walcott to place this animal amongst the Annelida. They are re-interpreted as gill bars throughout an extended pharynx. This specimen also shows the posterior bilobed structure (USNM 57630). (D, E) The proboscis, collar, trunk and gill bars are all apparent (USNM 57631). (F) The proboscis, circum-collar ridge and gill bars are extremely pronounced (USNM 57632). Direct light images: A, C, D, F; polarized light images: B, E. For acronyms, see Fig. 1. Scale bars: 10 mm. (JPG 8141 kb) [file 12915_2016_271_MOESM1_ESM.jpg]

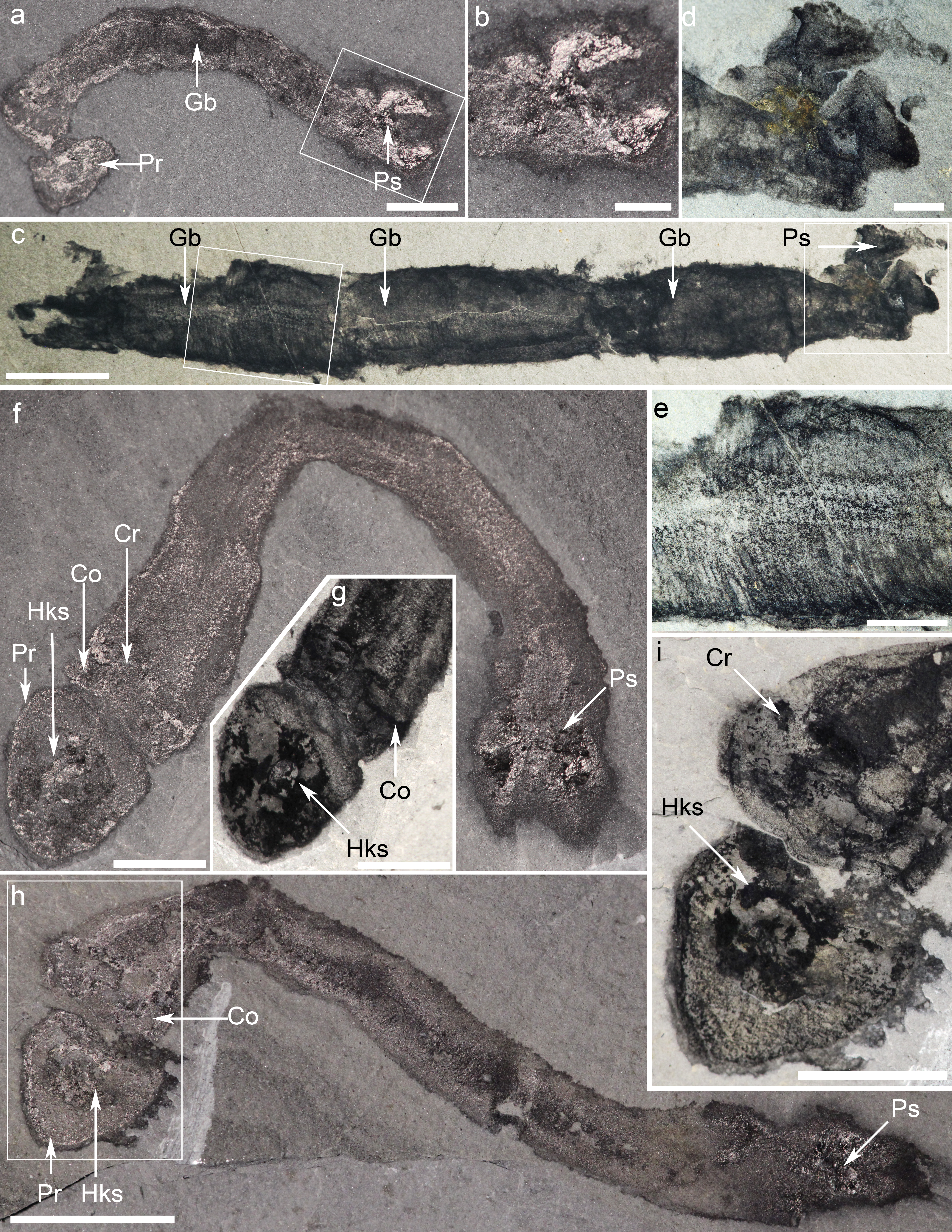

Supplement: Additional file 2: — Oesia disjuncta from the Burgess Shale (all specimens from Marble Canyon except USNM 203001, 203033 (C–E – Walcott Quarry). (A–B) Specimen showing the proboscis, gill bars, and exceptional preservation of the posterior bi-lobed structure (B = close-up of framed area in A) (ROM63714). (C–E) Nearly complete specimen — anterior region (to the left) missing; pharyngeal gill bars extending almost completely to the terminal end of the trunk and bilobed posterior structure (D, E = close-ups of framed areas in C) (USNM 203001, 203033). (F–G) Complete specimen showing rounded proboscis, collar, gill bars and bi-lobed posterior structure (ROM 63709). (H, I) Complete specimen showing rounded proboscis, kidney-heart-stomochord complex, collar, circum-collar ridge, gill bars and posterior structure. A patterning texture in the proboscis suggests possible proboscis muscles (ROM63707). Direct light images: A, F, H; Polarized light images: C, D, E, G, I. For acronyms see Fig. 1. Scale bars: A, C, F, G, =5 mm; B, D, E=1mm; H=10 mm; I=2 mm. (PNG 60109 kb) [file 12915_2016_271_MOESM2_ESM.png]

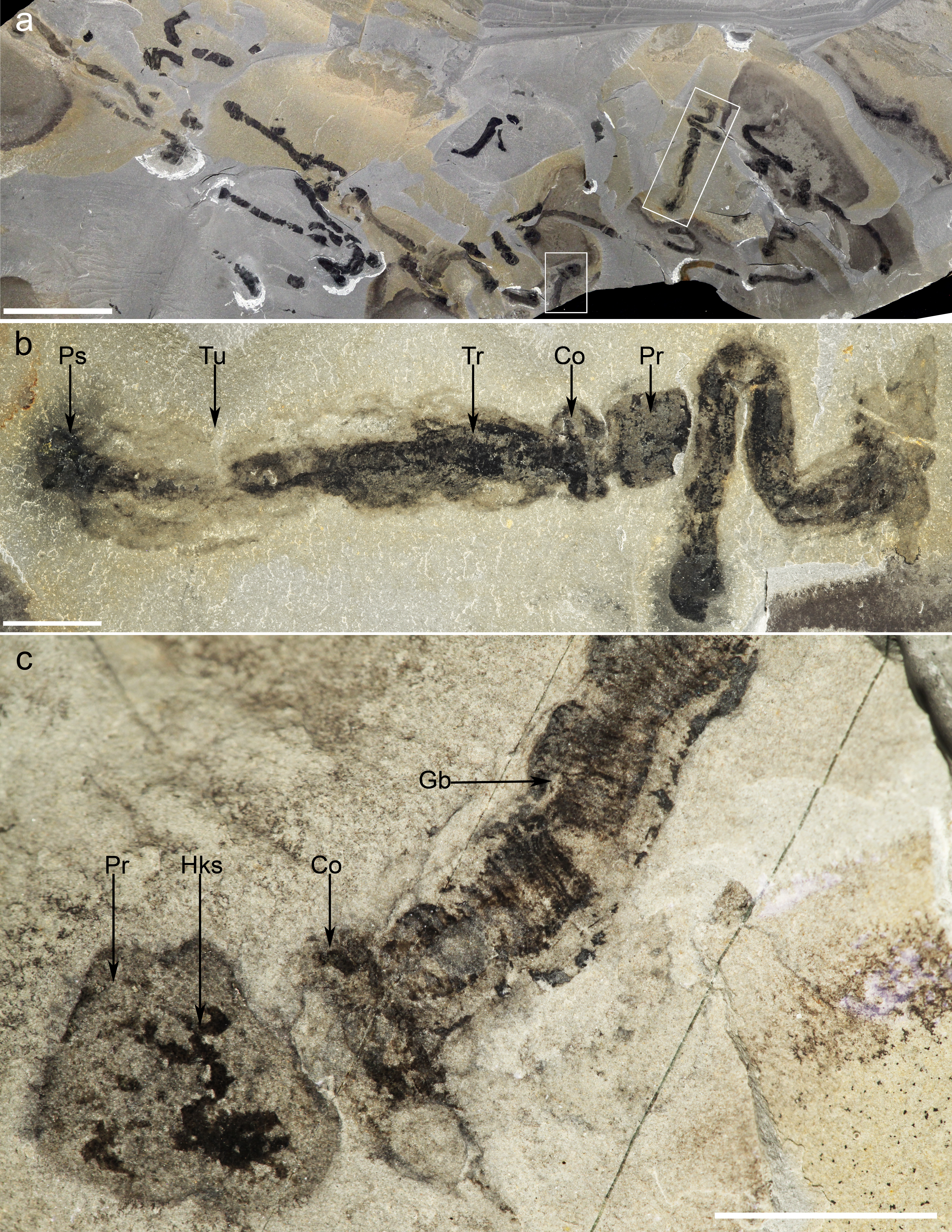

Supplement: Additional file 3: — Cluster of Oesia disjuncta from the Burgess Shale (Marble Canyon) preserved on the surface of one large slab (ROM 63735). O. disjuncta is highly gregarious at the Marble Canyon, occurring in high abundance across all stratigraphic levels (see also Additional file 7). (A) Overall slab. (B–C) Close-ups of framed areas in A. The specimen on the left in B is preserved in a decayed tube. The specimen in C shows tripartite body plan, gill bars and kidney-heart-stomochord complex. Polarized light images: A–C. Acronyms see Fig. 1. Scale bars: A = 10 cm; B, C = 1 cm. (PNG 59904 kb) [file 12915_2016_271_MOESM3_ESM.png]

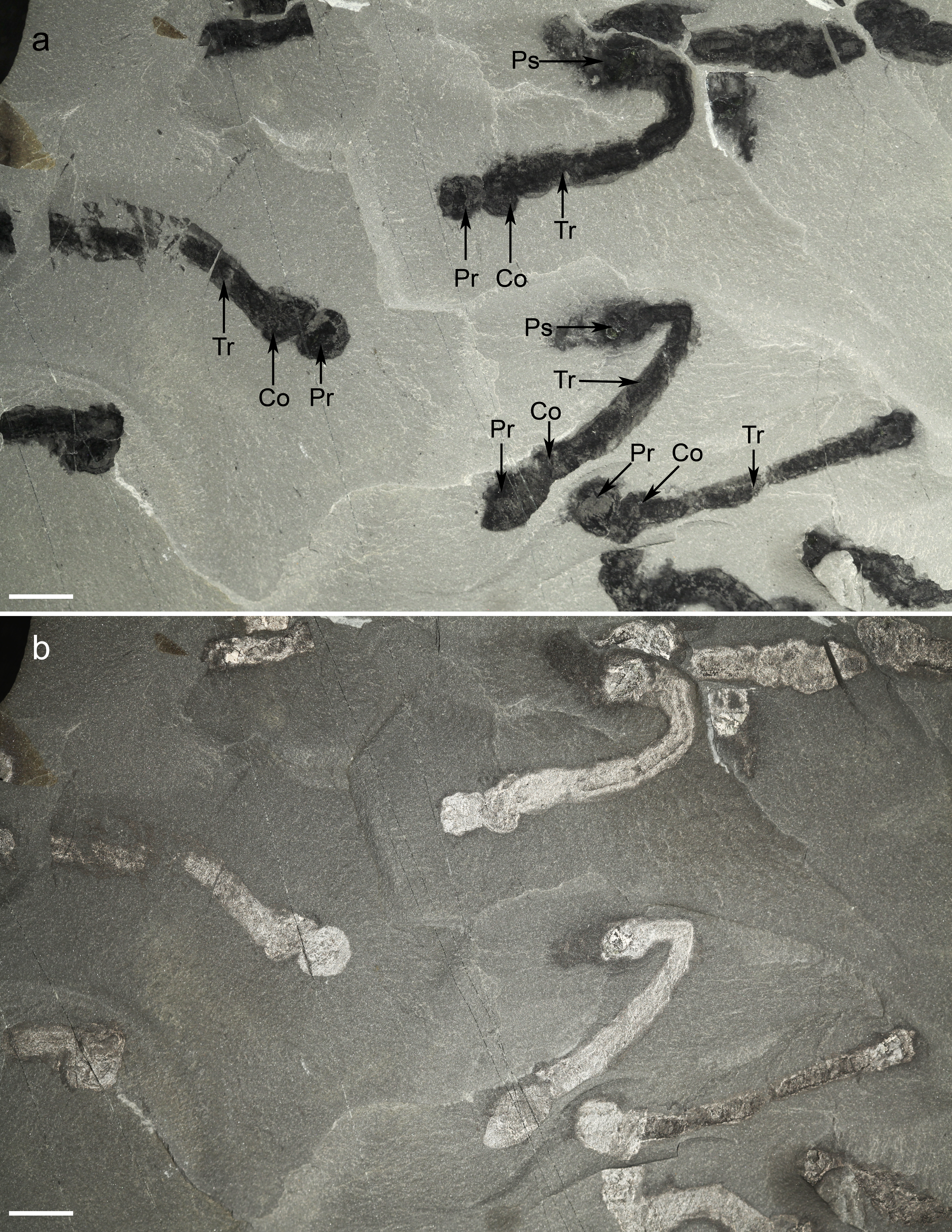

Supplement: Additional file 4: — Close up of a cluster of Oesia disjuncta from the Burgess Shale (Marble Canyon) preserved on the surface of one large slab (ROM 63736). Specimens show clear tripartite body plan as well as variation in proboscis size and shape. Direct light image: A; Polarized light image: B. For acronyms, see Fig. 1. Scale bars = 1 cm. (PNG 61235 kb) [file 12915_2016_271_MOESM4_ESM.png]

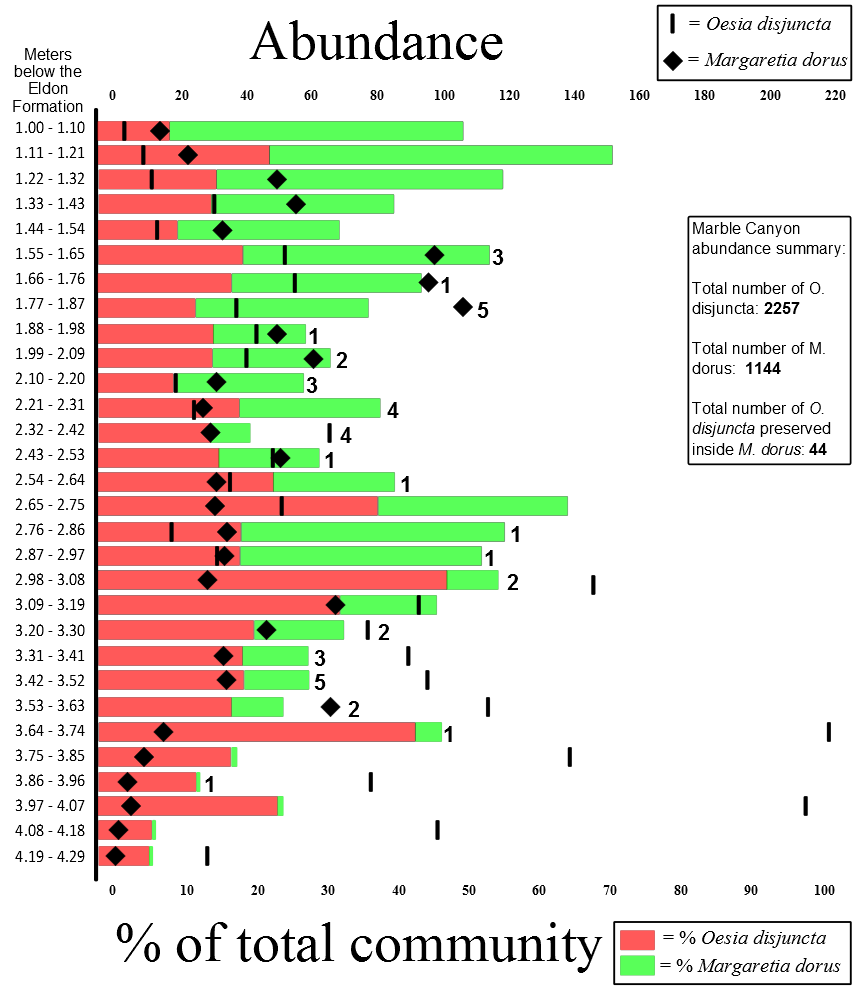

Supplement: Additional file 6: — Stratigraphic variation in abundance of Oesia disjuncta and Margaretia dorus in the Marble Canyon paleocommunity. Bars and diamonds indicate numerical abundances of each taxon across 10 cm stratigraphic bins. Coloured bars indicate the percentage of the total number of specimens found within that bin from O. disjuncta and M. dorus (total community size estimates from 2012 and 2014 field collections). Numbers next to the bars indicate the number of occurrences of Oesia preserved inside of Margaretia observed within that bin. Stratigraphic levels on the vertical axis represent negative meters from the boundary between the Eldon and Stephen Formations as a reference point. (PNG 62 kb) [file 12915_2016_271_MOESM6_ESM.png]

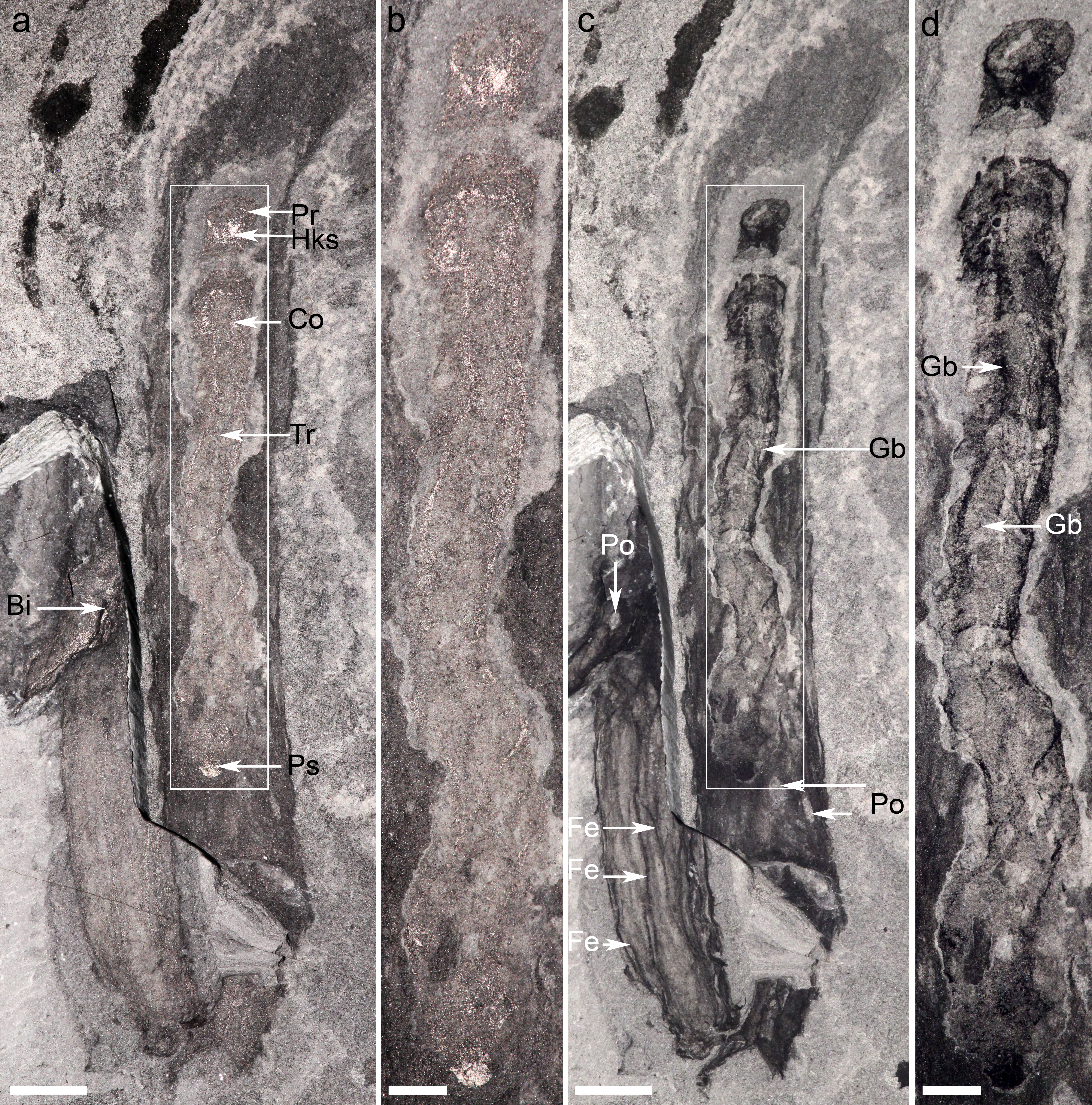

Supplement: Additional file 7: — Detailed imagery of ROM 63738 (see also Fig. 2d, e). Reflective areas in direct light tend to appear black using polarized light, emphasizing for example the kidney-heart-stomochord complex and the posterior structure (B, D are close-ups of framed areas in A and C). Direct light images: A, B; polarized light images: C, D. For acronyms see Fig. 1 and Fig. 2. Scale bars: A, C: 10 mm; B, D: 5 mm. (PNG 54067 kb) [file 12915_2016_271_MOESM7_ESM.png]

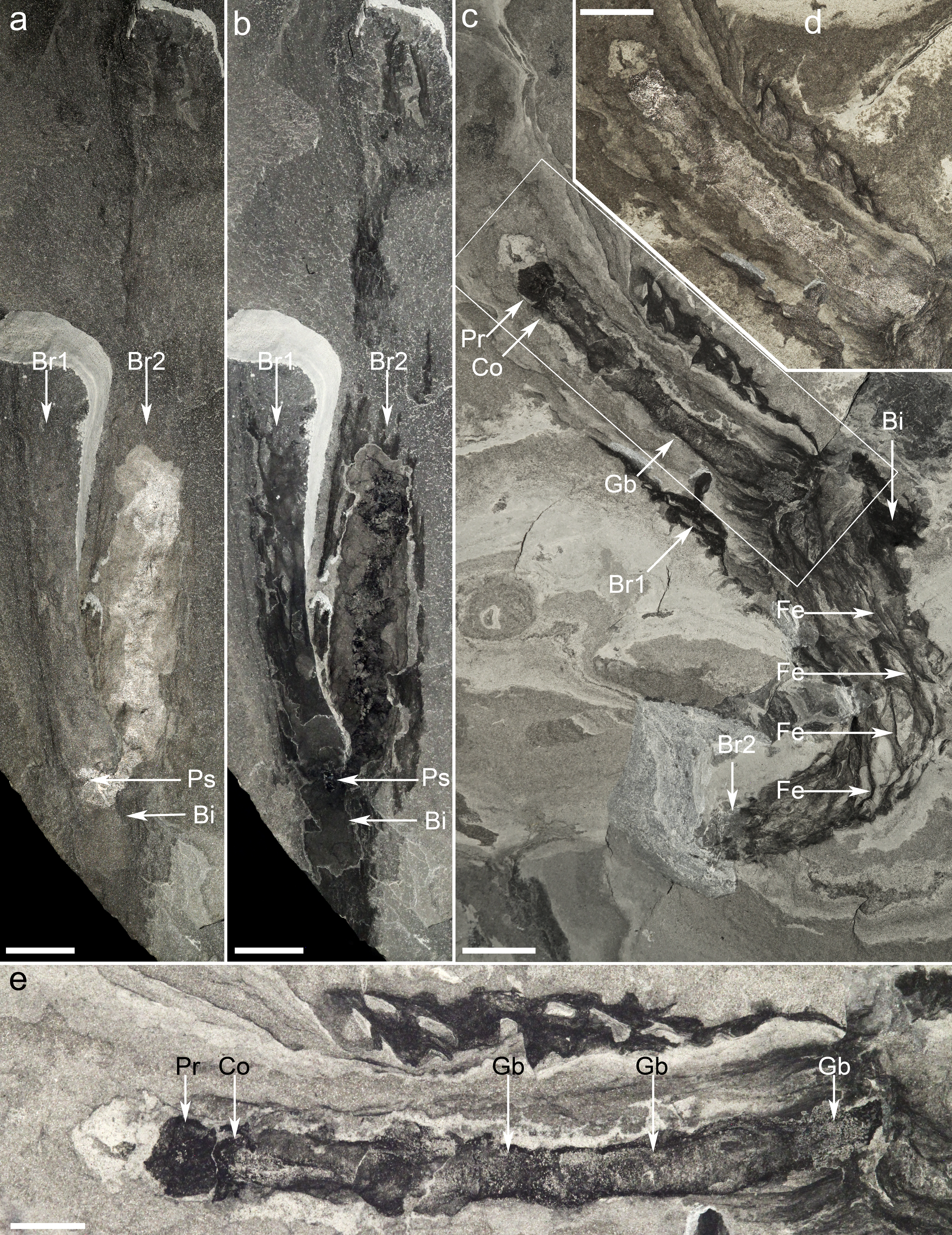

Supplement: Additional file 8: — Additional specimens of Oesia disjuncta preserved inside branching tubes from the Burgess Shale (Marble Canyon). The worms show a typical high degree of reflectivity in contrast with the surrounding tubes. (A, B) ROM 63712. (C–E) ROM 63708. Direct light images: A, D; polarized light images: B, C, E. Br1: branch 1, Br2: branch 2, other acronyms see Fig. 1 and Fig. 2. Scale bars: A, B, E: 5 mm, C, D = 10 mm. (PNG 64717 kb) [file 12915_2016_271_MOESM8_ESM.png]

*Spartobranchus tenuis*

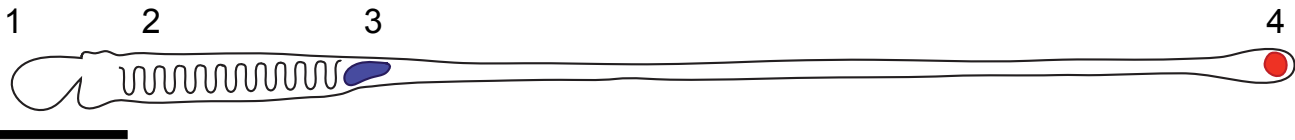

*Oesia disjuncta*

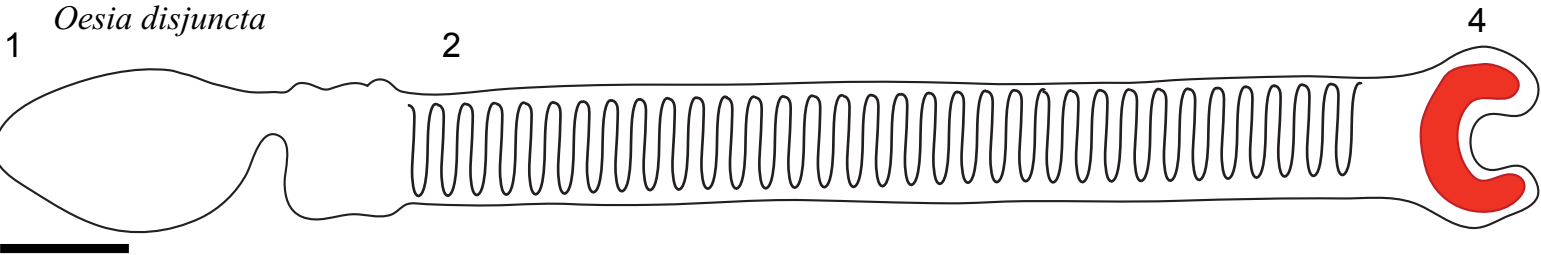

Tube of  
*S.*  
*tenuis*

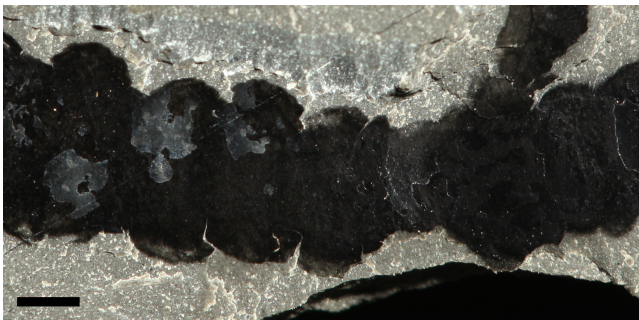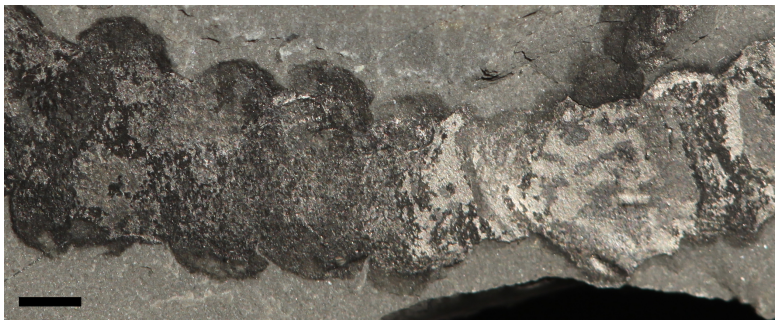

Supplement: Additional file 10: — Major morphological differences between the two Cambrian, tubicolous enteropneusts Spartobranchus tenuis (top box) and Oesia disjuncta (middle box): (1) S. tenuis is thin and elongate and the trunk much more variable in width compared to O. disjuncta, which is stout and does not vary in width across the length of the trunk; (2) the pharyngeal gill bars are restricted to approximately 10–20 % of the total trunk length [10] in S. tenuis, but extend approximately 80 % of the total trunk length in O. disjuncta; (3) S. tenuis possesses an esophageal organ while O. disjuncta does not; (4) S. tenuis has a bulbous terminal structure while O. disjuncta has a claw-shaped terminal apparatus; also the tube of S. tenuis has an externally corrugated but smooth texture with no evidence of pores or openings (bottom box; ROM 94189, while the tube of O. disjuncta is fibrous, much larger and has helicoidally arranged openings of variable sizes (not illustrated in this figure). Scale bars for the line drawings: 1 cm, scale bars for the tube: 1 mm. (PDF 7926 kb) [file 12915_2016_271_MOESM10_ESM.pdf]
